# Promoter DNA Methylation Metrics and Gene Expression in Human CD3+ T Cells: Sensitivity to Analytical Choices

**DOI:** 10.3390/genes17091033

**Published:** 2026-08-28

**Authors:** Ragıp Onur Öztornacı

**Affiliations:** Department of Biostatistics and Medical Informatics, Faculty of Medicine, Gaziantep University, 27310 Gaziantep, Türkiye; onur.oztornaci@gmail.com

**Keywords:** DNA methylation, CAMDA, methylation entropy, WGBS, promoter, gene expression, CpG island, analytical robustness, CD3+ T cells

## Abstract

**Background/Objectives**: Promoter DNA methylation is conventionally summarised as a regional mean. Two alternatives have been proposed to capture information the mean discards: the concurrence ratio derived from partially methylated bisulfite reads (CAMDA) and single-CpG Shannon (β-) entropy. Benchmarks of such metrics typically report one coefficient per metric on a single gene set at a single aggregation scale. We asked how far that comparison depends on analytical choices that are rarely reported. **Methods**: Matched whole-genome bisulfite sequencing (WGBS) and RNA-seq from one donor of human CD3+ T cells were analysed. Promoter windows spanning −1000 to +500 bp around the transcription start site (TSS) were divided into fifteen 100 bp bins; all three metrics were computed on an identical CpG set and correlated with log_2_(FPKM) by Spearman’s ρ. The comparison was repeated across annotation source, quantification level, coverage threshold, aggregation scheme, the treatment of zero-expression transcripts and CpG island status, with metric differences assessed by paired bootstrap over promoters and over chromosomes. **Results**: Across a grid of five analytical choices, the mean methylation coefficient ranged from −0.145 to −0.593 and the ordering of the metrics reversed between configurations. At the 100 bp bin scale, the largest difference between metrics was 0.037; aggregating the same CpGs to the whole promoter opened differences of up to 0.31, so the apparent superiority of a metric is itself a function of the aggregation scale. CAMDA exceeded the regional mean consistently but modestly, with the advantage largest in distal upstream bins and indistinguishable from zero adjacent to the TSS. β-Entropy is a strictly monotone function of the folded measure 1 − 2|β − 0.5|; at the 100 bp scale, the mean of per-CpG entropy tracked the folding transform of regional mean methylation to within 0.002 in every bin, while at the whole-promoter scale, the two separated by 0.041. Within CpG islands, the regional mean weakened (ρ = −0.246) while CAMDA did not (−0.494). **Conclusions**: In this single-donor dataset, analytical design affected the estimated coupling more than the choice of summary metric. The observed range is dataset-specific and should not be interpreted as a transferable numerical estimate. Rather, these results show that evaluating plausible analytical configurations can provide important context when comparing promoter methylation summary metrics.

## 1. Introduction

CpG methylation at gene promoters has been central to mammalian epigenetics since promoter methylation was first linked to transcriptional repression [1,2]. The picture that emerged, in which CpG island promoters at active genes are largely unmethylated while persistent methylation marks silent loci, has held across cell types and organisms [3,4], and whole-genome bisulfite sequencing (WGBS) reproduced it at base resolution in primary human samples [5,6]. The relationship is nonetheless weaker than the qualitative picture suggests: reported Spearman correlations between promoter methylation and expression in bulk tissue rarely exceed 0.3 in absolute value [7,8]. A regional mean conflates CpGs that are stably methylated, stably unmethylated and partially methylated, and the within-region distribution of states is lost when the average is taken [9,10].

Two families of alternatives have been proposed. The first treats methylation as a steady state set by opposing enzyme families: DNMT1, DNMT3A and DNMT3B add methyl groups, while TET dioxygenases oxidise 5-methylcytosine and initiate active demethylation [11,12]. Shi and colleagues used read-level co-occurrence to define the CAMDA concurrence ratio, the proportion of unmethylated CpGs within partially methylated bisulfite reads [13]. ChIP-seq atlases place TET1 at proximal CpG island promoters and DNMT3A more distally [14], and loss-of-function mutations in DNMT3A and TET2 co-occur in T-cell lymphoma [15,16], supporting a functional coupling between writer and eraser activities. The second family quantifies methylation heterogeneity by information-theoretic or epiallele-based measures [9,10,17,18], for which read-level entropy computed over four or more adjacent CpGs is the established formulation [19]. A single-CpG Bernoulli entropy H(β) = −β log_2_β − (1−β) log_2_(1−β) is sometimes used as an accessible proxy, since it is maximal at β = 0.5 and approaches zero at committed states. Comparisons of within-sample heterogeneity scores, nonetheless, treat read-level formulations as the reference [19].

Comparisons among these metrics are typically reported as one coefficient per metric, computed on one gene set, at one coverage threshold, at one aggregation scale, with the implicit claim that the resulting ordering is a property of the metrics [13,19]. That claim is rarely tested. Promoter methylation–expression correlations are known to depend on CpG density class [20], and the choice between transcript-level and gene-level quantification changes both the effective sample size and the weighting of multi-isoform loci. Whether a reported ranking survives these choices is an empirical question with practical consequences. Epigenome-wide association and methylation-QTL studies still rely mainly on single-CpG array probes [21], and their prioritisation depends on which metric and which positional window is believed to carry the strongest signal.

CD3+ peripheral-blood T cells suit the question. They are a lineage-defined population in which methylation at lineage-defining loci is stable [22,23]; matched WGBS and RNA-seq from the same donor and cell fraction are public [5]; and the same sample underlies the original CAMDA report [13], permitting direct comparison with a published benchmark. The aim is methodological rather than biological. We ask first how much the estimated strength of promoter methylation–expression coupling depends on decisions that are ordinarily reported in a single Methods sentence, if at all. These are transcript versus gene-level quantification, coverage threshold, annotation source, regional aggregation scheme, and the CpG island composition of the gene set. Second, we ask how large the differences between the three summary metrics are when all are computed on an identical CpG set, and where in the promoter window any difference is located. Because the comparison rests on a single donor and a single cell type, the results are presented as a benchmark of analytical sensitivity in one well-characterised dataset rather than as an estimate of population-level coupling.

## 2. Materials and Methods

### 2.1. Data

Matched WGBS and RNA-seq for CD3+ primary peripheral blood T cells were obtained from the NCBI Gene Expression Omnibus (WGBS: GSM1186660/SRR1104838; RNA-seq: GSM1220574/SRR980468), both on hg19, from a single 37-year-old male donor. Annotation was GENCODE v19 [24] for the primary analysis and NCBI RefSeq [25], obtained as the UCSC refGene table [26], for the annotation-sensitivity analysis. RNA-seq was quantified with RSEM [27] and reported as FPKM. Because Spearman’s ρ is rank-based, it is invariant under monotone per-sample transformation, so the FPKM versus TPM choice does not affect the reported coefficients.

### 2.2. Methylation Metrics

WGBS reads were aligned with BSMAP [28] and per-CpG tables generated with the reference CAMDA implementation [13], which emits the concurrence ratio and the conventional methylation ratio in a common format. Mean methylation was β = C/CT, where C is the methylated read count and CT the coverage. CAMDA was computed as in Shi et al. [13]. Two coverage thresholds were used: CT ≥ 10, conventional in WGBS analysis [5,6], and CT ≥ 4, matching the original CAMDA report. Filters were applied identically to both tables so that all metrics rest on the same set of sites. Strand orientation was preserved when defining the TSS.

Single-CpG β-entropy was computed as H(β) above, with H(0) = H(1) = 0 by the limiting value. This departs deliberately from a common implementation choice. Evaluated directly at β ∈ {0,1}, the expression is indeterminate, and discarding the resulting missing values removes precisely those promoters whose CpGs are uniformly committed. In the present data, that convention removed roughly 40% of promoter bins and rendered the entropy tables non-comparable with the other metrics. Retaining these sites keeps all three metrics on an identical CpG and promoter set. This single-CpG quantity is not read-level epiallele entropy [9,10,17,19], which requires phased multi-CpG reads. It is examined here because it appears in applied work as a heterogeneity proxy, and one aim of this study is to test whether that use is justified.

### 2.3. Promoter Binning and Aggregation

For each transcript, a −1000 to +500 bp window around the strand-corrected TSS was partitioned into 15 non-overlapping 100 bp bins. The bin value was the unweighted mean of per-CpG values.

Two regional aggregation schemes were compared for whole-promoter summaries. Let *C*_i_ and *CT*_i_ denote the methylated and total read counts at CpG *i*, let *A*_i_ and *AT*_i_ denote the corresponding concurrence counts, and let *k* be the number of CpGs surviving the coverage filter in a region. Pooled aggregation weights each CpG by its coverage, giving Σ*C*_i_/Σ*CT*_i_ for mean methylation and Σ*A*_i_/Σ*AT*_i_ for CAMDA. CpG-weighted aggregation, as used in the original report [13], takes the unweighted mean of the per-CpG ratios, (1/*k*) Σ (*C*_i_/*CT*_i_) and (1/*k*) Σ (*A*_i_/*AT*_i_). The two are not equivalent when coverage varies within a region.

Entropy has no pooled counterpart of this form, since it is not a ratio of counts. Two distinct regional quantities, therefore, exist and are reported separately: the mean of the per-CpG entropies, (1/*k*) Σ *H*(β_i_), and the entropy evaluated at the regional mean, *H*[(1/*k*) Σ β_i_]. Averaging a non-linear function is not the same as applying it to an average, so agreement between these two quantities depends on the aggregation scale. CpGs failing the coverage filter are excluded before aggregation under every scheme, and a region is retained only if the surviving CpGs meet the minimum-count threshold, so all three metrics are always computed on an identical CpG set within a region.

Because the number of promoters passing a per-bin CpG threshold varies systematically with position, CpG density being highest close to the TSS [1,20], two complementary analyses were run. The primary analysis used all promoters passing a ≥10 CpG per-bin threshold, giving bin-specific sample sizes. A confirmatory analysis was restricted to the fixed set of promoters with data in all fifteen bins, so that positional comparisons are made on identical promoters throughout the window.

### 2.4. CpG Islands

CpG island coordinates were taken from the UCSC hg19 cpgIslandExt track [26] (30,344 islands), which implements the Gardiner-Garden and Frommer criteria [29]. A promoter was classified as island-associated if its TSS fell within an annotated island; 65.2% of promoters were island-associated under RefSeq gene-level annotation. This definition was chosen because the metrics are correlated with expression at the TSS-anchored window, and island status of the TSS itself is the property expected to constrain the dynamic range of the regional mean. It necessarily excludes promoters whose window overlaps an island that does not contain the TSS. Because that choice affects the reported proportion, both definitions were compared in a separate sensitivity analysis.

### 2.5. Statistics

Spearman’s ρ was computed between each bin-level metric and log_2_(FPKM). It was preferred over Pearson’s r because both variables are markedly non-normal [30]. Because promoters sharing a locus are not independent and the correlations being compared share a common outcome, differences between metrics were assessed by paired bootstrap over promoters with 2000 replicates, reporting observed Δ|ρ| with percentile 95% confidence intervals [31]. Tests assuming independent samples, including Fisher’s r-to-z transformation, are not valid for dependent overlapping correlations of this kind [32] and were not used. Given per-bin sample sizes in the tens of thousands, interpretation rests on the magnitude of ρ and Δ|ρ| rather than on *p*-values [30]. Promoters resampled independently are not fully exchangeable, since promoters on the same chromosome share chromatin domains and co-expression structure [5,21]. The bin-level comparison was therefore repeated under a block bootstrap in which chromosomes rather than promoters were resampled to preserve within-chromosome dependence. The analysis involves many bin by metric by configuration comparisons. Interpretation therefore rests on the consistency of the pattern across bins and configurations rather than on any individual coefficient, and every Δ|ρ| is reported with its interval so that the whole profile can be judged rather than its extremes.

### 2.6. Sensitivity Grid

The whole-promoter comparison was repeated across a grid of five binary choices. These are annotation (GENCODE v19 versus RefSeq), quantification level (transcript versus gene), coverage threshold (≥4× versus ≥10×), regional aggregation (pooled versus CpG-weighted), and the treatment of zero-expression transcripts (dropped, with log_2_ FPKM, versus retained, with log_2_(FPKM + 0.1)). The treatment of zero-expression transcripts is included as a factor in its own right rather than varied alongside others, because excluding those transcripts conditions on the outcome. One annotation-by-quantification-level combination is not estimable, and because the remaining three factors are crossed within it, this accounts for eight of the thirty-two cells that five binary factors would otherwise define. RNA-seq was quantified against the GENCODE v19 transcriptome, so transcript-level abundances exist for GENCODE accessions but not for RefSeq ones; RefSeq therefore enters only at the gene level, where isoform abundances are summed to the locus. The design is consequently crossed over all estimable combinations, 24 cells, rather than fully crossed. Marginal effects were therefore estimated on common support. Under gene-level quantification, the representative transcript was selected in three ways: the highest-FPKM isoform, the longest transcript, and a canonical rule preferring curated RefSeq (NM_) entries and otherwise the longest transcript. The first is outcome-dependent and the other two are not; all three are reported. Aggregation formulas are defined explicitly for each metric.

### 2.7. Availability

All analysis code is available at https://github.com/onurslash/promoter-dnam-metrics (accessed on 26 August 2026).

## 3. Results

### 3.1. Single-CpG β-Entropy Adds Little Beyond the Regional Mean

At the single-CpG level, H(β) is a strictly monotone function of the folded methylation measure 1 − 2|β − 0.5|; the two, therefore, have identical rank correlation with any outcome by construction. H(β) is not monotone in β itself, being symmetric about β = 0.5. Whether the identity survives regional aggregation is not automatic, because the two regional quantities are different objects: the bin-level mean of per-CpG entropy values on one hand, and the folding transform applied to the bin-level mean methylation on the other. Averaging a non-linear function is not the same as applying it to an average. Empirically, the two coincided closely at the 100 bp scale. Across all fifteen bins, the correlation of the mean of the per-CpG entropies with expression matched that of the folding transform applied to the bin mean to within 0.002 (Table 1). At this resolution, the Jensen gap introduced by aggregation is therefore negligible, and single-CpG β-entropy adds essentially nothing to the regional mean as a predictor of expression. This equivalence is specific to the 100 bp scale and does not persist across the whole-promoter window.

### 3.2. Positional Profile: Coupling Is Strongest Downstream of the TSS

**Figure 1:**
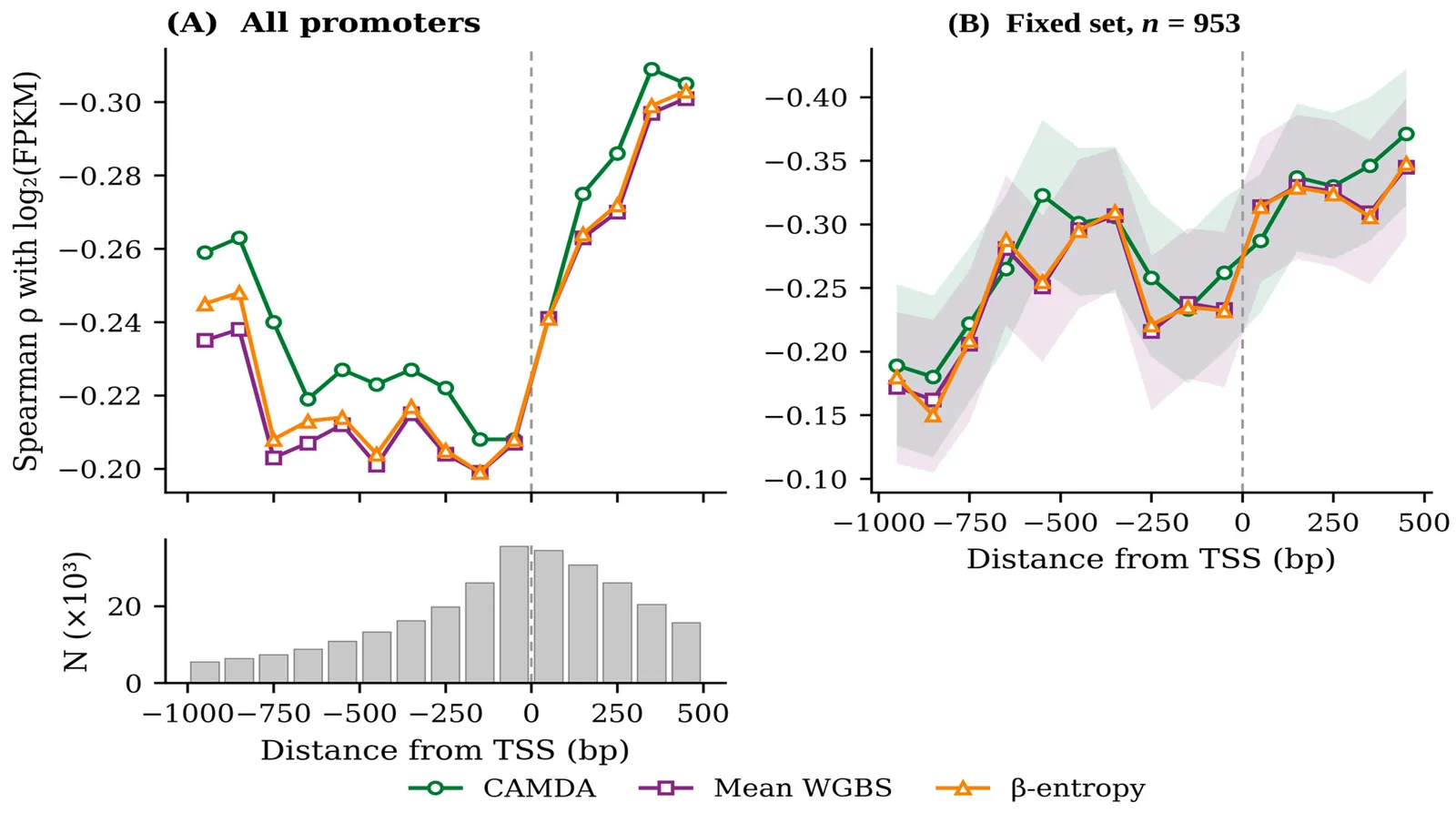
Bin-level Spearman correlation between each methylation metric and log_2_(FPKM) across the −1000 to +500 bp promoter window, in fifteen 100 bp bins plotted at bin midpoints. The dashed line marks the transcription start site. (**A**) All promoters passing the per-bin CpG threshold, with the number contributing to each bin shown below; this number varies with position. (**B**) The same analysis restricted to the 953 promoters with data in all fifteen bins, so that positional comparisons are made on identical promoters throughout the window. Within each bin, all three metrics are computed on the identical CpG set. The shaded areas in panel B represent percentile 95% confidence intervals based on 2000 bootstrap replicates. Shaded bands in panel B show percentile 95% confidence intervals from 2000 bootstrap replicates.

All three metrics were negatively associated with expression in every bin (Table 2). The profile is not the monotone strengthening toward the TSS that a core-promoter model would predict [1,3]. Correlations were moderate in the distal upstream bins and weakest in a shallow trough spanning roughly −200 to 0 bp. They were strongest downstream: at +400 to +500 bp, mean methylation reached ρ = −0.301 and CAMDA ρ = −0.305, compared to ρ ≈ −0.20 at the TSS itself (Figure 1A).

Because per-bin sample size varies with position (5450 promoters in the most distal bin compared to 35,520 near the TSS), this shape could in principle reflect changing promoter composition rather than position. Restricting the analysis to the 953 promoters with data in all fifteen bins reproduced it (Figure 1B). For CAMDA, correlations rose from −0.189 (95% CI −0.253 to −0.126) in bin 1 to −0.371 (−0.422 to −0.315) in bin 15, with the same local trough around the TSS. Percentile intervals from 2000 bootstrap replicates are shown as shaded bands in Figure 1B; the intervals for the most distal and most downstream bins do not overlap, so the positional difference is not attributable to sampling variability in this smaller set. The downstream maximum is therefore a positional feature, not a composition artefact, although the fixed set is small and restricted to unusually CpG-dense promoters.

### 3.3. CAMDA’s Advantage over the Mean Is Consistent but Small

**Figure 2:**
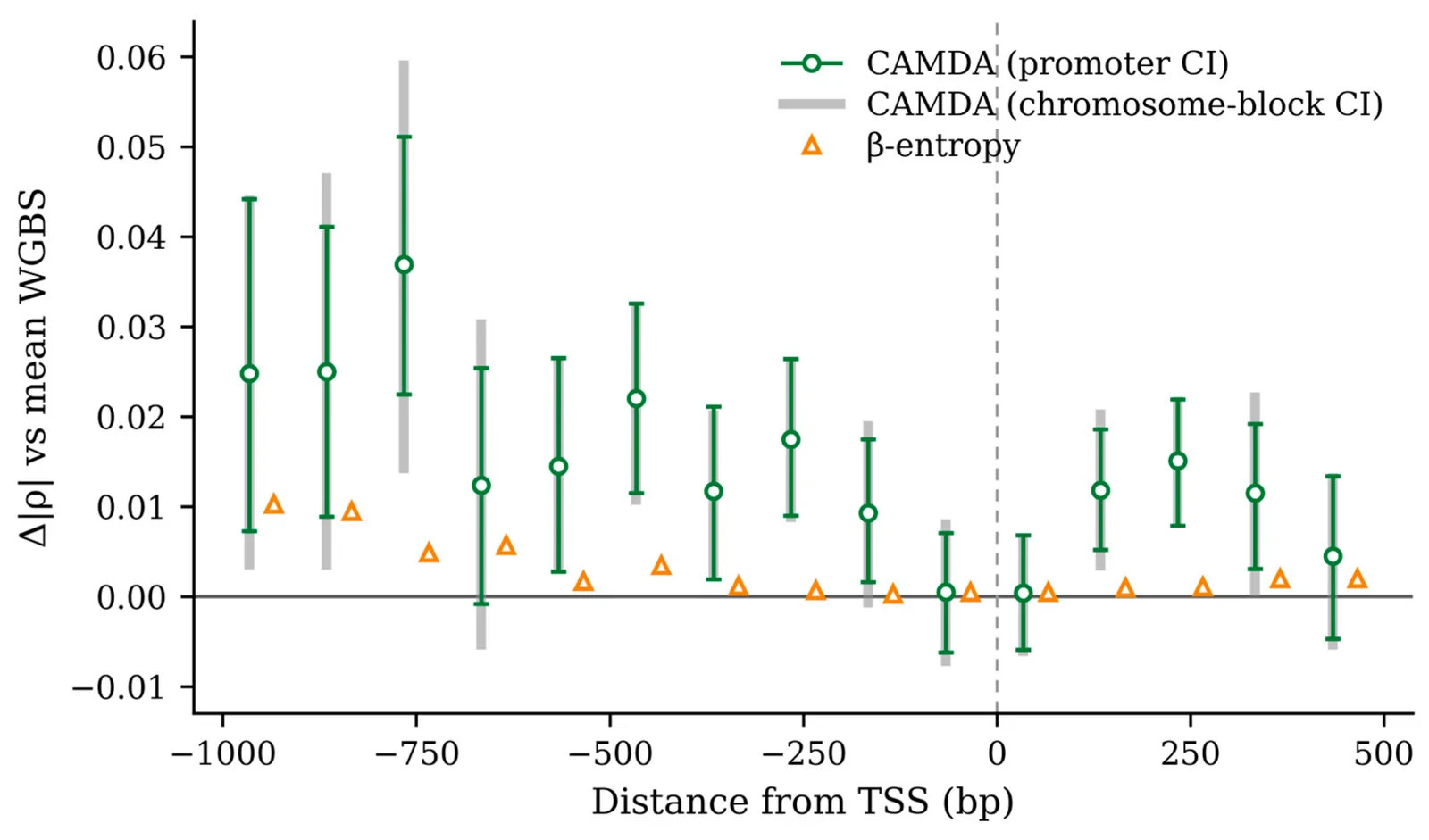
Paired bootstrap comparison of CAMDA and β-entropy against mean WGBS methylation. Points show Δ|ρ|, the absolute correlation of the metric minus that of mean methylation in the same bin; positive values favour the metric. Percentile 95% confidence intervals from 2000 replicates are shown for CAMDA under both resampling units: the narrow bars resample promoters, while the wide grey bars resample chromosomes. β-Entropy values are plotted without intervals because they lie within 0.010 of zero throughout.

A paired bootstrap comparison against mean methylation (Table 3, Figure 2) shows CAMDA ahead in every bin, with intervals excluding zero in 11 of 15. The margin is small. The largest Δ|ρ| was 0.037 at bin 3, and in the two bins flanking the TSS, the difference was indistinguishable from zero (Δ|ρ| = 0.0005 and 0.0004). β-Entropy differed from mean methylation by Δ|ρ| ≤ 0.010 in every bin.

The location of CAMDA’s advantage is informative. It is largest in the distal upstream bins, where mean methylation has the least dynamic range because most non-island CpGs there are near-fully methylated regardless of expression [6,20]. It is smallest at the TSS, where the two metrics coincide. The advantage, therefore, does not follow the pattern that writer–eraser competition at the core promoter would predict [14].

### 3.4. Analytical Choices Move the Coefficients Further than Metric Choice Does

**Figure 3:**
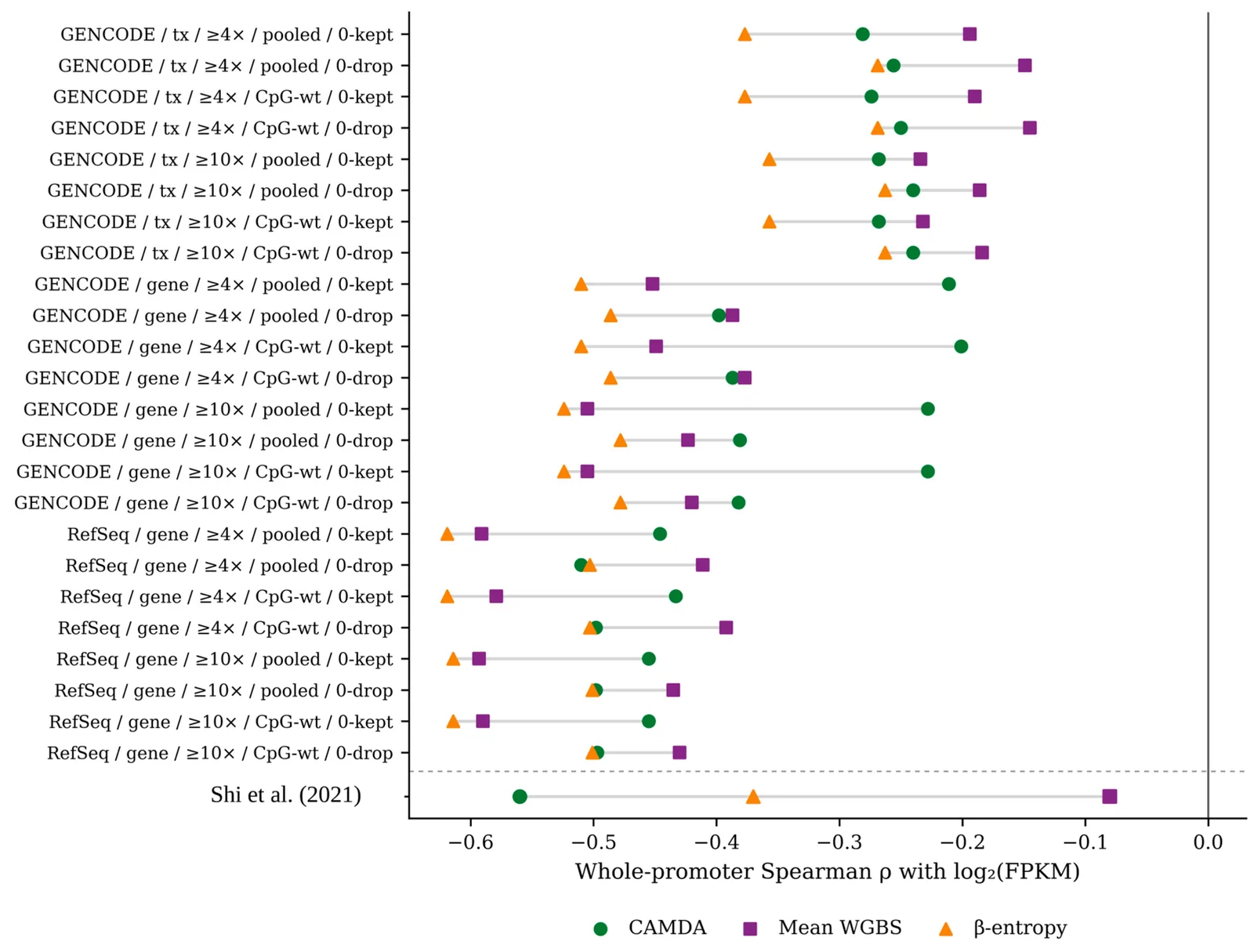
All 24 estimable configurations of the sensitivity grid. Each row represents one combination of annotation source, quantification level, coverage threshold, regional aggregation and the treatment of zero-expression transcripts; the grey connector spans the extreme metric estimates in that configuration. The bottom row, below the dashed rule, reports the values published by Shi et al. (2021) for the same WGBS sample [13].

Repeating the whole-promoter comparison across the 24 estimable configurations shows that both the coefficients and the ordering of the metrics are contingent on analytical decisions (Table 4, Figure 3). Each choice can be estimated with the others averaged over, rather than inferred from configurations that differ in several respects at once (Table 5). Because RefSeq enters only at the gene level, the level and annotation contrasts are estimated on common support: the level effect within GENCODE, where both levels exist, and the annotation effect within gene-level analysis, where both annotations exist. The remaining three factors are balanced across the grid and are averaged over all 24 cells. For mean methylation, the quantification level has the largest marginal effect, moving |ρ| by 0.250 between transcript- and gene-level analysis; the treatment of zero-expression transcripts contributes 0.098, annotation source 0.063, coverage threshold 0.035 and aggregation scheme 0.006. The ordering is not the same for every metric. For β-entropy the level effect is again largest at 0.183, but for CAMDA the annotation effect (0.172) exceeds the level effect (0.042), and the treatment of zero-expression transcripts moves CAMDA in the opposite direction to the other two metrics. No single choice, therefore, has the largest influence uniformly.

The ordering is not preserved across the grid. Mean methylation was the strongest of the three in eight configurations, β-entropy in fifteen and CAMDA in one. Under GENCODE gene-level annotation at ≥10× coverage with zero-expression transcripts retained, mean methylation reached −0.505, while CAMDA reached only −0.228. Under RefSeq gene-level annotation at ≥4× with those transcripts dropped, CAMDA (−0.510) exceeded mean methylation (−0.411). These configurations differ only in choices typically reported in a single Methods sentence, if at all. Across the grid, mean methylation spans −0.145 to −0.593, a range of 0.448, against the largest between-metric difference at fixed configuration of 0.310.

The magnitude and ordering of these effects varied by metric. No single analytical choice had the largest influence across all three metrics, and the sensitivity of the estimated association depended on the configuration being evaluated.

### 3.5. The Gene-Level Definition Is Not Driven by the Expression-Based Selection Rule

Selecting the highest-FPKM isoform for each gene uses the same expression values that are subsequently correlated with methylation, and could in principle inflate the transcript versus gene contrast. Two expression-independent rules were therefore compared against it (Table 6). Under GENCODE annotation at ≥4× coverage with CpG-weighted aggregation, the mean methylation coefficient was −0.377 with the highest-FPKM rule and −0.327 with either the longest-transcript or the canonical rule; the corresponding CAMDA values were −0.387 and −0.377. The comparison is informative only under GENCODE. Because RNA-seq was quantified against the GENCODE transcriptome, RefSeq transcripts of the same locus inherit the same summed gene-level abundance, so a highest-FPKM rule cannot discriminate among them and resolves ties by annotation order rather than by expression. The RefSeq rows of Table 6 are reported for completeness, but the three rules there are close to one another for that reason, not because selection is immaterial.

Under GENCODE, the selection rule therefore accounts for roughly 0.05 of the coefficient, while the transcript-to-gene change accounts for approximately 0.18 under the same annotation and aggregation. The outcome-dependent rule therefore exaggerates the contrast but does not create it. Gene-level results elsewhere in this manuscript use the highest-FPKM rule for comparability with the published benchmark; the expression-independent values are given here so that the difference remains visible.

### 3.6. The Equivalence Between the Two Entropy Forms Holds at 100 bp but Not Across the Whole Promoter

Within 100 bp bins, the mean of the per-CpG entropies and the folding transform applied to the bin mean gave indistinguishable rank correlations. Whether that holds at coarser resolution is a separate question, since the Jensen gap between averaging a non-linear function and applying it to an average grows with the heterogeneity of the values being averaged.

**Figure 4:**
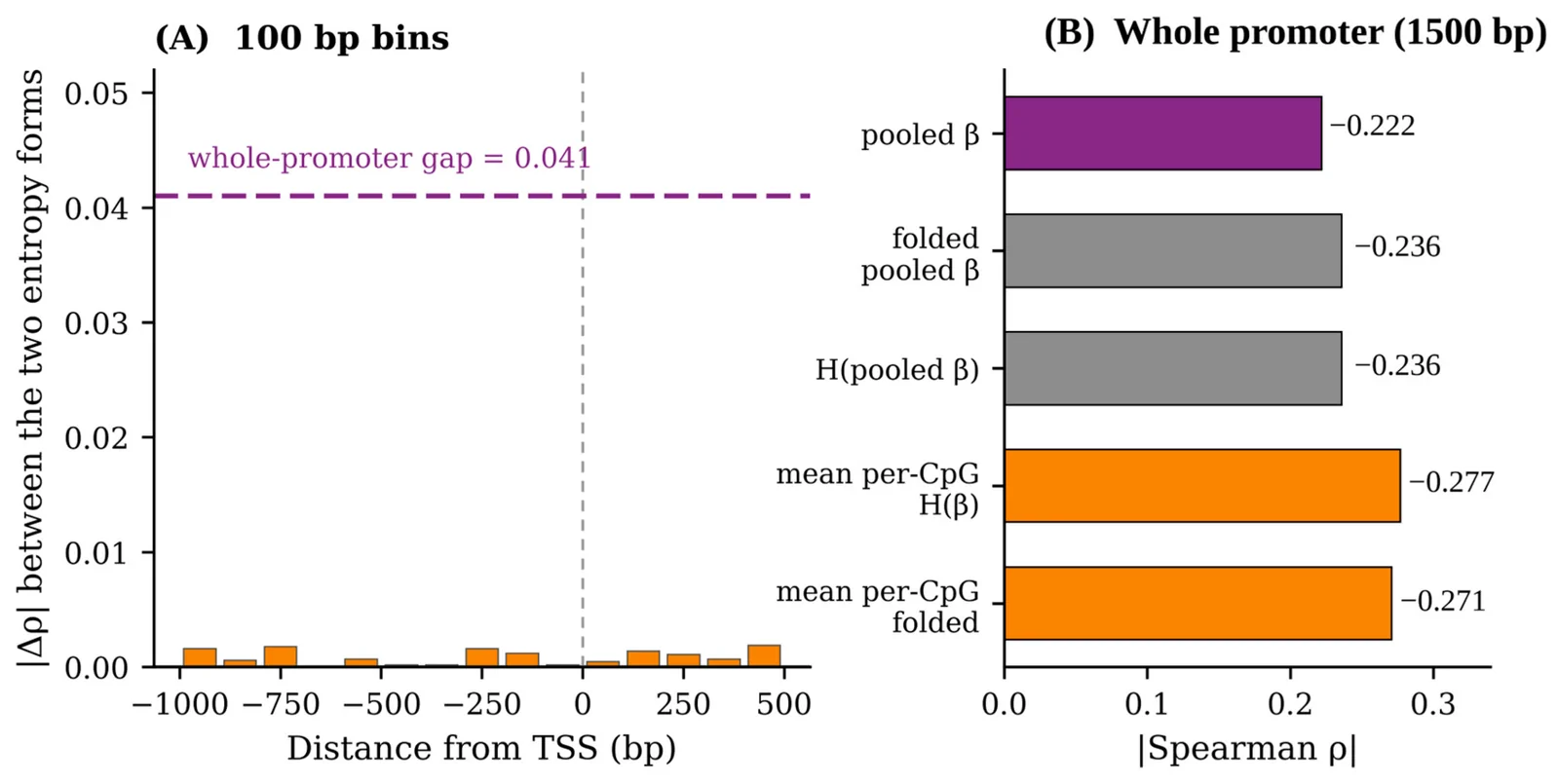
Aggregation scale governs whether the two regional entropy forms differ. (**A**) Absolute difference in Spearman correlation with expression between the mean of the per-CpG entropies and the folding transform applied to the bin mean, within each 100 bp bin. The dashed line marks the corresponding difference when the same CpGs are aggregated over the whole 1500 bp promoter window. (**B**) Whole-promoter correlations for five related regional quantities computed on the same CpG set.

It does not hold at the whole-promoter scale (Figure 4). Within 100 bp bins, the gap between the two quantities was at most 0.002 in every bin. Aggregating the same CpGs over the full −1000 to +500 bp window increased it to 0.041. The mean of the per-CpG entropies gave ρ = −0.277, against −0.236 for both the folding transform of the pooled mean and for the entropy evaluated at the pooled mean; the pooled mean itself gave −0.222 (≥10× coverage, zero-expression transcripts dropped, *n* = 64,620). A 1500 bp promoter window contains CpGs at widely different methylation levels, while a 100 bp bin generally does not, and the non-linearity of H therefore has room to act at the coarser scale.

The whole-promoter sensitivity grid appeared to contradict this: there, β-entropy, computed as the mean of per-CpG values, was the strongest of the three metrics in fifteen of 24 configurations, while at the bin level it is indistinguishable from the mean. The two results are the same comparison at two aggregation scales. Whether one metric carries information beyond another therefore depends on the scale at which the region is summarised. Results obtained at a single aggregation scale may not generalise to other regional resolutions.

### 3.7. CpG Island Composition Explains Part of the Discrepancy with the Published Benchmark

Under the configuration closest to the published benchmark, CAMDA reproduced reasonably closely (−0.498 versus −0.56). Mean methylation did not: we obtained −0.393 where −0.08 was reported. Because the published gene set was restricted to promoters detectable by all three of its metrics, including a read-level entropy measure requiring reads spanning at least four CpGs, that set is expected to be enriched for high-CpG-density, island-associated promoters.

**Figure 5:**
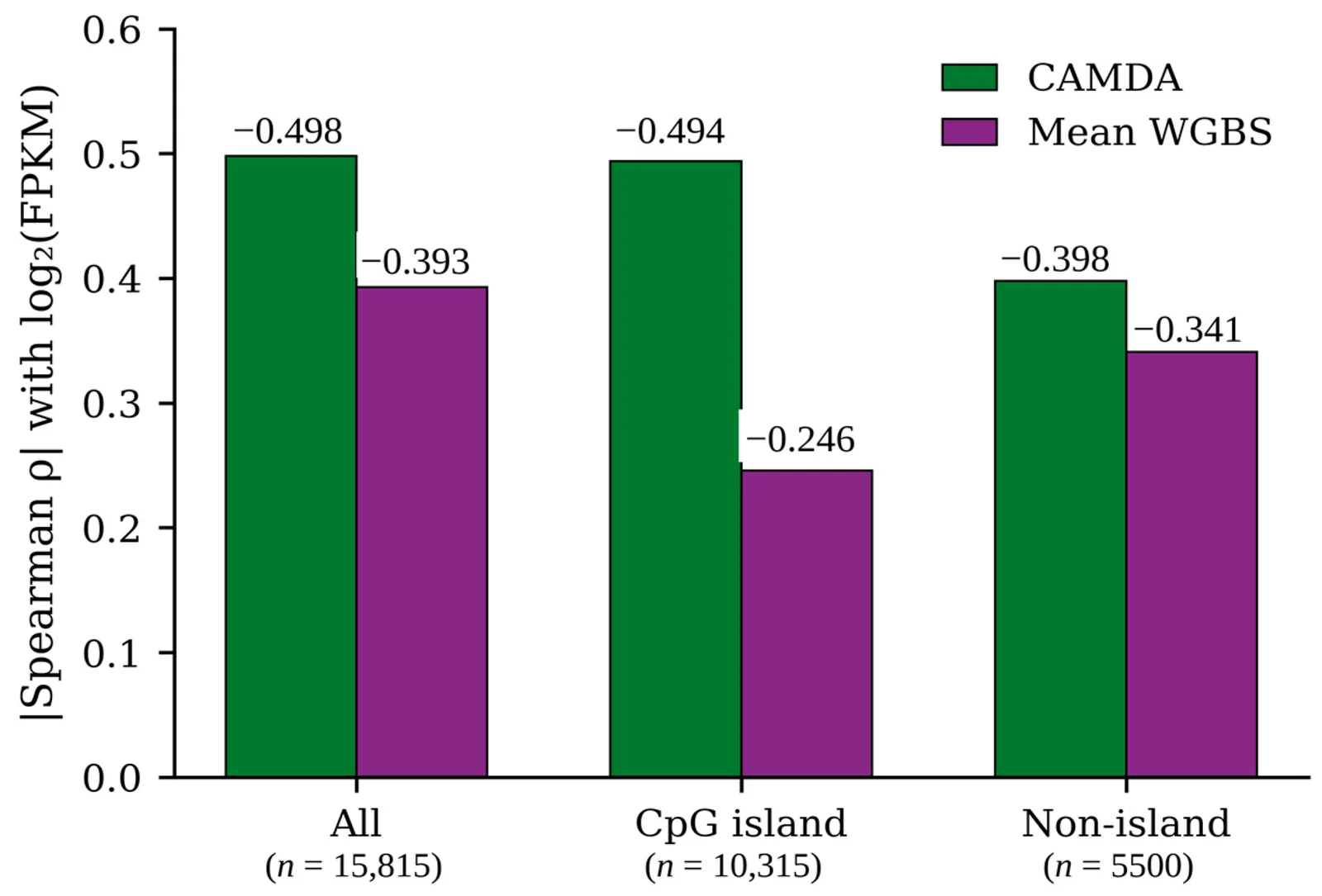
Whole-promoter correlations stratified by CpG island status of the transcription start site, under RefSeq gene-level annotation, ≥4× coverage and CpG-weighted aggregation. Bars show |ρ|; signed values are printed above each bar.

Stratification by island status is consistent with this but does not fully account for the gap (Table 7, Figure 5). Within island-associated promoters, mean methylation weakened from −0.393 to −0.246, while CAMDA was essentially unchanged at −0.494; outside islands, the two converged. CpG islands at active promoters are uniformly hypomethylated [1,3], which compresses the dynamic range of the regional mean and mechanically limits the correlation it can attain. The concurrence ratio is bounded away from its ceiling in both states and is not compressed in the same way. The direction is as predicted, but −0.246 remains well above the published −0.08, so island composition explains part rather than all of the difference. Two further explanations were tested directly; neither accounted for the remaining gap.

At the single-CpG level, the two metrics are themselves related: across 26.5 million CpGs passing the ≥4× filter, the Spearman ρ between CAMDA and β was −0.543. CAMDA was zero at 58.7% of sites, with a median of 0.000 against a median β of 0.857. A concurrence measure behaves this way by construction: it is bounded toward zero at committed sites and peaks at intermediate methylation [13]. That is why the two metrics separate at island promoters, where β is uniformly low, and converge elsewhere.

### 3.8. Robustness of the Bootstrap Scheme, Coverage and the CpG Island Definition

Three additional analyses were run to test assumptions underlying the results above.

**Bootstrap scheme.** Resampling promoters treats them as exchangeable, which they are not: promoters on the same chromosome share chromatin domains and regulatory programmes. Repeating the bin-level comparison with chromosomes as the resampling unit widened the intervals slightly and reduced the number of bins whose interval excluded zero from 11 to 10 of 15 (Table 8). The point estimates are unchanged by construction. The conclusion that CAMDA exceeds the regional mean by a small margin, absent adjacent to the TSS, is therefore not an artefact of treating promoters as independent draws.

**CpG density and coverage.** The gene set of the published benchmark was restricted to promoters measurable by a read-level entropy score, which requires reads spanning four or more adjacent CpGs [13,19]. Phased reads are not recoverable from per-site tables, so two proxies for that restriction were examined. Raising the minimum number of covered CpGs per promoter moved the mean methylation coefficient from −0.378 at one CpG to −0.312 at fifty, with 14,823 promoters remaining at the strictest threshold (Table 9; GENCODE gene-level, zero-expression transcripts dropped). Extrapolating to the published set size of 9876 would not approach −0.08. Requiring every CpG in the window to reach 4× coverage left only 62 promoters, so the published gene set cannot be reconstructed from this sequencing run at all.

Sequencing depth acts in the opposite direction to the one assumed. Promoters in the lowest tertile of median coverage gave ρ = −0.268, compared to −0.393 in the highest; stratifying instead by the proportion of CpGs reaching 4× gave ρ = −0.195, −0.409 and −0.367 across tertiles. Raising the per-CpG coverage threshold strengthened the coefficient monotonically from −0.396 at 4× to −0.436 at 10×, declining thereafter only as the sample shrank. Sparse coverage therefore attenuates the association rather than inflating it, and a deeper dataset would be expected to yield a stronger coefficient than ours, not one closer to zero. Neither CpG density restriction nor sequencing depth explains the residual discrepancy, which we therefore leave unresolved.

**CpG island definition.** Classifying a promoter as island-associated when its window overlaps an island, rather than when its TSS lies inside one, raised the proportion from 78.9% to 94.1% in the transcript-level set used here. The stratified pattern remained unchanged in direction: the regional mean was weaker inside islands and stronger outside under both definitions, while CAMDA varied little between strata (Table 10). The overlap definition sharpened the contrast, with the non-island mean coefficient falling to −0.167. The proportions differ from 65.2% because that value was obtained from the RefSeq gene-level set with FPKM > 0, whereas this comparison uses the transcript-level set retaining zero-expression promoters.

## 4. Discussion

In this dataset, the estimated strength of promoter methylation–expression coupling depended more on analytical configuration than on the summary metric. Across the grid, the mean methylation coefficient varied from −0.145 to −0.593, a range of 0.448, whereas the largest between-metric difference within a fixed whole-promoter configuration was 0.310. At the 100 bp bin scale, the largest between-metric difference was 0.037. Thus, the relative influence of analytical choices and metric selection depended on the aggregation scale. The observed spread is specific to this dataset and is not expected to reproduce across donors or cell types, where CpG density composition, sequencing depth and isoform structure differ. The general conclusion is qualitative: analytical choices can materially alter the estimated association and are important to report when comparing methylation summary metrics.

Single-CpG β-entropy, which appears in applied work as a heterogeneity measure, is a monotone transform of the folded methylation measure rather than of β itself, and its bin-level rank correlation with expression was indistinguishable from that of the regional mean. Regional aggregation did not separate the two: the difference between the mean of the per-CpG entropies and the folding transform of the bin mean was below 0.002 in every bin. At the 100 bp scale, reporting single-CpG entropy alongside mean methylation as though they were distinct metrics, therefore, adds little. At the whole-promoter scale, the two do separate, by 0.041, but this separation arises from non-linear averaging over a heterogeneous window rather than from independent biological information, and it appears only once the region is large enough for methylation levels within it to differ substantially. Read-level epiallele entropy [9,10,17,19] is computed over phased multi-CpG reads and is a different quantity; nothing here bears on it, except that the single-CpG proxy cannot substitute for it.

The concurrence ratio added information, but less than a single-configuration benchmark might suggest. Its margin over the regional mean was at most 0.037 in absolute correlation and vanished at the TSS. The advantage was most evident in distal upstream bins and depended on CpG island composition. The regional mean was roughly a third weaker within CpG islands than outside, whereas CAMDA changed little. CpG islands at active promoters are uniformly hypomethylated [1,3], which limits the dynamic range of mean methylation. Accordingly, the size of the CAMDA–mean difference depends on gene-set composition. Coverage structure also influenced mean methylation: sparse or uneven coverage attenuated the coefficient by roughly a third across tertiles, consistent with measurement error. In this dataset, CAMDA was less sensitive than mean methylation to CpG island composition, but the observed advantage was much smaller than the published contrast of −0.56 versus −0.08.

The positional profile also departs from expectations. Rather than strengthening monotonically toward the TSS, coupling was weakest in a shallow trough around the TSS and strongest in the +300 to +500 bp bins, a pattern reproduced on a fixed promoter set. Most of that interval falls within the first exon, and first-exon methylation has been reported to track silencing more tightly than upstream promoter methylation [33]. Nucleosome architecture is a plausible additional contributor, since the +1 nucleosome typically sits +60 to +200 bp downstream of the TSS [34,35]. The data are consistent with a first-exon mechanism but cannot establish one.

The broader implication concerns how metric comparisons are conducted. Comparisons are easier to interpret when the promoter window and gene set are defined before selecting the summary statistic and when sensitivity to transcript versus gene and island versus non-island definitions is reported. This separates variation attributable to analytical configuration from variation associated with the biological signal being summarised.

Several limitations bound what can be concluded. The analysis rests on a single donor and a single CD3+ T-cell dataset. The sensitivity pattern reported here is therefore a property of this dataset under these configurations. Neither the absolute coefficients nor the relative ordering should be assumed to transfer to other cell types, other donors, or population-level designs. Matched WGBS and RNA-seq of comparable depth exist for other primary haematopoietic populations [5], and extending the grid across them would be required before any general claim could be made. The fixed-promoter confirmatory analysis retained only 953 promoters, a CpG-dense minority, so its positional profile carries wide uncertainty even though it agrees with the primary analysis. Sequencing depth from the single run analysed here may differ from that available to the original report, and concurrence measures are depth-sensitive because they depend on observing partially methylated reads [13]. The effect of sequencing depth on the residual CAMDA difference was not directly quantified here. For mean methylation, however, the two candidate explanations that could be tested—CpG density restriction and coverage—did not account for the discrepancy, and increasing coverage strengthened rather than attenuated the coefficient. The mean methylation discrepancy therefore remains unresolved and would require the phased read-level data underlying the original gene set to address fully. The β-entropy studied here is explicitly the single-CpG proxy, not read-level epiallele entropy, and the conclusions drawn about it do not extend to the latter. Finally, the coefficients reported are positional associations, not causal effect sizes; whether coupling at any bin is direct or mediated by allele-specific methylation [21] is not resolvable without genotype data.

## 5. Conclusions

In matched WGBS and RNA-seq from a single donor of human CD3+ T cells, the estimated strength of promoter methylation–expression coupling was more sensitive to analytical design than to the choice of summary metric. Across the grid of analytical configurations, the mean methylation coefficient ranged from −0.145 to −0.593, and the ordering of the three metrics reversed between configurations. At the whole-promoter scale, the largest difference between metrics at any fixed configuration was 0.310; at the 100 bp bin scale, it was 0.037, so the comparison between analytical and metric influence itself depends on the resolution at which it is made. The CAMDA concurrence ratio outperformed the regional mean consistently but modestly. Its advantage was concentrated in distal upstream bins and was indistinguishable from zero at the transcription start site, which points to the regional mean’s dependence on CpG island composition rather than on any property localised at the TSS. Single-CpG β-entropy is a monotone transform of the folded methylation measure and added essentially nothing beyond the regional mean at either the single-CpG or the bin level, which does not bear on read-level epiallele entropy. Coupling was strongest downstream of the TSS, in the first-exon window, rather than at the TSS itself. These observations come from one donor and one cell type. The magnitude of the sensitivity reported here is specific to this dataset and is not offered as a general quantity. These findings indicate that sensitivity analyses across plausible analytical configurations can provide useful context when benchmarking promoter methylation summary metrics.

## Figures and Tables

**Table 1:** Bin-level Spearman correlations with log_2_(FPKM) for two distinct regional quantities: the mean of the per-CpG entropies and the folded measure 1 − 2|β − 0.5| applied to the bin-level mean methylation. Bin 1 spans −1000 to −900 bp; bin 15 spans +400 to +500 bp. The two columns agree to within 0.001 throughout.

Bin	Position (bp)	ρ, Mean per-CpG Entropy	ρ, Folded Bin Mean
1	−1000/−900	−0.245	−0.244
3	−800/−700	−0.208	−0.207
5	−600/−500	−0.214	−0.215
7	−400/−300	−0.217	−0.216
9	−200/−100	−0.199	−0.200
10	−100/0	−0.208	−0.208
11	0/+100	−0.241	−0.242
12	+100/+200	−0.264	−0.264
13	+200/+300	−0.272	−0.271
14	+300/+400	−0.299	−0.299
15	+400/+500	−0.303	−0.302

**Table 2:** Bin-level Spearman correlations between each metric and log_2_(FPKM). *n* is the number of promoters contributing to each bin; all three metrics are computed on the identical CpG set within a bin. Eleven of fifteen bins are shown.

Bin	Position (bp)	*n*	CAMDA	Mean WGBS	β-Entropy
1	−1000/−900	5450	−0.259	−0.235	−0.245
3	−800/−700	7341	−0.240	−0.203	−0.208
5	−600/−500	10,849	−0.227	−0.212	−0.214
7	−400/−300	16,181	−0.227	−0.215	−0.217
9	−200/−100	26,064	−0.208	−0.199	−0.199
10	−100/0	35,520	−0.208	−0.207	−0.208
11	0/+100	34,435	−0.241	−0.241	−0.241
12	+100/+200	30,700	−0.275	−0.263	−0.264
13	+200/+300	26,070	−0.286	−0.270	−0.272
14	+300/+400	20,452	−0.309	−0.297	−0.299
15	+400/+500	15,654	−0.305	−0.301	−0.303

**Table 3:** Paired bootstrap comparison of each dynamic metric against mean WGBS methylation. Δ|ρ| is the absolute correlation of the metric minus that of mean methylation in the same bin; positive values favour the dynamic metric. Percentile 95% confidence intervals from 2000 replicates resampling promoters; the corresponding chromosome-block intervals are given in Section 3.8.

Bin	Position (bp)	Δ|ρ| CAMDA	95% CI
1	−1000/−900	0.0248	0.0073 to 0.0442
3	−800/−700	0.0369	0.0225 to 0.0511
6	−500/−400	0.0220	0.0115 to 0.0326
8	−300/−200	0.0175	0.0090 to 0.0264
10	−100/0	0.0005	−0.0062 to 0.0071
11	0/+100	0.0004	−0.0059 to 0.0068
13	+200/+300	0.0151	0.0079 to 0.0219
15	+400/+500	0.0045	−0.0047 to 0.0134

**Table 4:** Whole-promoter Spearman correlations across the sensitivity grid. Selected configurations from the 24-cell design are shown; the complete grid appears in Figure 3 and in the repository. “0-kept” retains zero-expression transcripts under log_2_(FPKM + 0.1); “0-drop” excludes them. The final row reports values published for the same WGBS sample [13].

Annotation	Level	Cov.	Aggregation	*n*	CAMDA	WGBS	Entropy
GENCODE v19	transcript	≥4×	pooled	95,142	−0.256	−0.150	−0.222
GENCODE v19	transcript	≥10×	pooled	85,519	−0.241	−0.186	−0.232
GENCODE v19	gene	≥4×	pooled	24,927	−0.399	−0.388	−0.372
GENCODE v19	gene	≥10×	pooled	22,507	−0.382	−0.424	−0.399
RefSeq	gene	≥4×	CpG-weighted	15,815	−0.498	−0.393	—
Shi et al. [13]	gene	≥4×	CpG-weighted	9876	−0.56	−0.08	−0.37

**Table 5:** Marginal effect of each analytical choice on |ρ|, estimated with the remaining choices averaged over. Positive values indicate that the first-named level of the contrast gives the larger absolute correlation. * Estimated within GENCODE annotation, where both quantification levels exist. † Estimated within gene-level quantification, where both annotations exist. The remaining three factors are balanced across all 24 cells.

Analytical Choice	Mean WGBS	CAMDA	β-Entropy
Quantification level (gene vs. transcript) *	0.250	0.042	0.183
Annotation (RefSeq vs. GENCODE) †	0.063	0.172	0.060
Zero-expression transcripts (kept vs. dropped)	0.098	−0.066	0.083
Coverage threshold (≥10× vs. ≥4×)	0.035	0.000	−0.005
Aggregation (CpG-weighted vs. pooled)	−0.006	−0.005	0.000

**Table 6:** Whole-promoter coefficients under three rules for choosing a representative transcript per gene (≥4× coverage, CpG-weighted aggregation, zero-expression transcripts dropped). The first row gives the transcript-level analysis for reference. Under RefSeq, the highest-FPKM rule is not discriminating, because all RefSeq transcripts of a locus inherit the same gene-level abundance from the GENCODE-based quantification; those rows are reported for completeness only.

Annotation	Representative Transcript	*n*	Mean WGBS	CAMDA
GENCODE v19	all transcripts	95,127	−0.145	−0.250
GENCODE v19	highest FPKM	24,912	−0.377	−0.387
GENCODE v19	longest	24,970	−0.327	−0.377
GENCODE v19	canonical	24,970	−0.327	−0.377
RefSeq	highest FPKM	15,815	−0.392	−0.498
RefSeq	longest	15,831	−0.375	−0.486
RefSeq	canonical	15,829	−0.377	−0.486

**Table 7:** Whole-promoter correlations stratified by CpG island status of the TSS (RefSeq gene-level, ≥4× coverage, CpG-weighted aggregation).

Promoter Stratum	*n*	CAMDA	Mean WGBS
All	15,815	−0.498	−0.393
CpG island (65.2%)	10,315	−0.494	−0.246
Non-island	5500	−0.398	−0.341

**Table 8:** Effect of the resampling unit on the CAMDA versus mean methylation comparison. Point estimates are identical; only the intervals differ. Selected bins shown.

Bin	Position (bp)	Δ|ρ|	95% CI, Promoter	95% CI, Chromosome
1	−1000/−900	0.0248	0.0073 to 0.0442	0.0030 to 0.0446
3	−800/−700	0.0369	0.0225 to 0.0511	0.0137 to 0.0596
4	−700/−600	0.0124	−0.0008 to 0.0254	−0.0059 to 0.0308
9	−200/−100	0.0093	0.0016 to 0.0175	−0.0012 to 0.0195
10	−100/0	0.0005	−0.0062 to 0.0071	−0.0077 to 0.0086
11	0/+100	0.0004	−0.0059 to 0.0068	−0.0066 to 0.0070
15	+400/+500	0.0045	−0.0047 to 0.0134	−0.0059 to 0.0137
Bins excluding 0	—	—	11 of 15	10 of 15

**Table 9:** Effect of CpG density and coverage restrictions on the whole-promoter mean methylation coefficient (GENCODE v19 gene-level, highest-FPKM representative transcript, zero-expression transcripts dropped). Published benchmark: *n* = 9876, ρ = −0.08.

Restriction	Threshold	*n*	ρ, Mean WGBS
Minimum CpGs at ≥4×	1	24,927	−0.378
	10	21,877	−0.392
	30	17,161	−0.363
	50	14,823	−0.312
All CpGs at ≥4×	≥4 CpG	62	—
Median promoter coverage	lowest tertile	10,214	−0.268
	highest tertile	6776	−0.393
Per-CpG coverage threshold	≥4×	23,982	−0.396
	≥10×	19,909	−0.436

**Table 10:** Sensitivity of the CpG island stratification to the definition of island association (transcript-level set, zero-expression promoters retained).

Definition	Stratum	*n*	CAMDA/Mean WGBS
TSS inside island (78.9%)	island	59,291	−0.271/−0.259
	non-island	15,853	−0.329/−0.268
Window overlaps island (94.1%)	island	70,747	−0.283/−0.269
	non-island	4397	−0.323/−0.167

## Data Availability

All data analysed are publicly available from the NCBI Gene Expression Omnibus under accessions GSM1186660 (WGBS, SRR1104838) and GSM1220574 (RNA-seq, SRR980468). CpG island coordinates were obtained from the UCSC hg19 cpgIslandExt track; gene annotation from GENCODE v19 and UCSC refGene. The complete analysis workflow, comprising the download and alignment script, a Quarto document that reproduces every table and figure, and the figure-generation code, is available at https://github.com/onurslash/promoter-dnam-metrics (accessed on 26 August 2026).
